# Prospective Case Series to Determine the Feasibility, Diagnostic Yield and Safety of Performing EBUS Guided Trans‐Bronchial Mediastinal Cryobiopsy (EBUS‐TBMC) of Lymph Nodes Using 1.1 mm Cryoprobe Through a Working Channel of Linear EBUS Bronchoscope Versus Conventional En‐Bloc Cryobiopsy

**DOI:** 10.1002/rcr2.70784

**Published:** 2026-10-14

**Authors:** Jolyn Ng, David Manners

**Affiliations:** ^1^ Department of Respiratory and Sleep Medicine St John of God Midland Public and Private Hospital Midland Western Australia Australia

**Keywords:** en‐block cryobiopsy, linear EBUS, through‐working‐channel cryobiopsy, trans‐bronchial mediastinal cryobiopsy

## Abstract

EBUS‐guided trans‐bronchial mediastinal cryobiopsy (EBUS‐TBMC) improves diagnostic yield over EBUS‐TBNA but conventionally requires en‐bloc removal of bronchoscope and frozen specimen, risking loss of airway control and procedural inefficiency. Retrieval of cryobiopsy specimens through‐working channel (TWC) may address these limitations, but its feasibility and diagnostic adequacy are uncertain. Prospective evaluation was completed of 13 patients undergoing EBUS‐TBMC at a single centre (August 2025 to January 2026). A 1.1 mm Erbe cryoprobe was advanced through an echobronchoscope with a 2.2 mm working channel (*BF‐UC190F*, Olympus Medical, Japan) via needle tract formed via EBUS‐TBNA. Fourteen lymph node stations underwent paired en‐bloc and TWC biopsies. Paired biopsies were completed in 92.3% of targets. Median specimen size was equivalent (3 mm each; *p* = 0.197). Diagnostic yield was 93% (en‐bloc) versus 79% (TWC); *p* = 0.48; versus 46% (EBUS‐TBNA). There were no post procedural complications. TWC cryobiopsy using a 1.1 mm probe through a standard 2.2 mm working channel is feasible and safe compared to conventional *en‐bloc* cryobiopsy.

## Graphical abstract

We describe the feasibility and diagnostic adequacies of performing cryobiopsies through the working channel (TWC) of a linear EBUS bronchoscope as compared to conventional en‐bloc cryobiopsy, which involves the en‐bloc removal of the linear bronchoscope, cryoprobe and frozen specimen. This case series demonstrates the feasibility of TWC cryobiopsies with comparable diagnostic yield to en‐bloc cryobiopsies, with no complications.

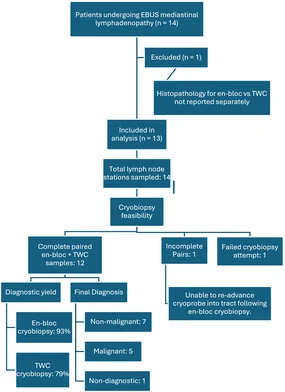

## 1 Introduction

EBUS‐TBMC has emerged as a technique capable of obtaining larger, architecturally preserved tissue samples compared with EBUS TBNA for both malignant and benign mediastinal lymphadenopathy. Reported diagnostic yield of EBUS‐TBMC approaches 91.9%, compared with 76.6% for EBUS‐TBNA alone [1, 2]. EBUS‐TBMC allows larger and intact tissue samples to be obtained, while preserving cellular architecture and enabling histopathology [3].

Current practice requires *en‐bloc* removal of the echobronchoscope with a 2.2 mm working channel (*BF‐UC190F*, Olympus Medical, Japan), cryoprobe and attached frozen specimen because frozen samples typically exceed the 2.2 mm working channel diameter of standard echobronchoscope with a 2.2 mm working channel (*BF‐UC190F*, Olympus Medical, Japan) [1]. While effective, *en‐bloc* removal has several limitations [4] including (a) loss of airway control during removal and re‐intubation, delaying recognition and management of bleeding, (b) repeat vocal cord passage, potentially increasing laryngeal irritation, (c) difficulty re‐navigating to the prior biopsy tract, especially in large or heterogeneous targets and (d) procedural inefficiency due to repeat scope removal and reinsertion.

Retrieving cryobiopsy specimens through the working channel could maintain continuous airway visualisation, avoid re‐intubations and streamline workflow. However, this approach may reduce specimen size and compromise histological adequacy. This prospective case series evaluates the feasibility, diagnostic yield and safety of performing EBUS‐TBMC using a 1.1 mm cryoprobe via the echobronchoscope with a 2.2 mm working channel (*BF‐UC190F*, Olympus Medical, Japan), compared with conventional *en‐bloc* cryobiopsy.

## 2 Case Series

### 2.1 Study Design and Population

Consecutive cases of patients undergoing EBUS‐TBMC for mediastinal and/or hilar lymphadenopathy between August 2025 and January 2026 were collected prospectively with retrospective analysis. Ethic approval was provided as part of a larger cohort of patients undergoing EBUS procedures at our institution (St John of God Healthcare Human Research Ethics Committee #1941, approval date 21 April 2022).

### 2.2 Cryobiopsy Technique

A 1.1 mm Erbe cryoprobe was advanced through a needle tract created using either Olympus Vizishot 2 Flex 22G or Mediglobe SonoTip TopGain 22G CrownTip needle. Two sets of two passes of cryobiopsies for each method was performed for each lymph node station [5]. The bronchoscopist attempted alternating methods of cryobiopsy removal, first through‐working channel (TWC), then *en‐bloc* removal of bronchoscope cryobiopsy. As it was not a comparative study with the different needle tips, the ease of cryobiopsies with varying needle tips could not be determined. The freeze‐time for both approaches was 3 s. Specimens were placed into separate formalin filled histopathology pots for processing. Failure definitions include ‘Failed Cryobiopsy’ with inability to advance the cryoprobe through the tract or failure to retrieve visible frozen tissue and ‘Incomplete Pair’ where only one method successfully obtained a sample.

### 2.3 Outcomes

Primary outcomes assessed were feasibility of completion of paired biopsies, specimen sizes (mm) and diagnostic yield as per strict ATS criteria [6]. Measurement of specimen sizes was performed by different anatomical pathologists in a non‐blinded manner and derived by the authors from the histopathology report. Median and range of specimen sizes were determined. Secondary outcomes included diagnostic yield upon comparison with EBUS‐TBNA and complications including bleeding, pneumothorax and pneumomediastinum assessed on post procedural chest x‐ray.

### 2.4 Statistical Analyses

Statistical analyses were performed on Microsoft Excel version 16.112.1. Wilcoxon signed‐rank test for specimen size comparison and McNemar's test for paired diagnostic yield were completed for lymph node stations with completed paired samples for continuous and dichotomous variables respectively. Overall diagnostic yield was calculated and stratified by malignant and benign disease.

### 2.5 Primary Outcomes

EBUS‐TBMC was performed on 14 patients and 13 were included in the analysis (Figure 1), with exclusion of a case due to non‐separated histopathology processing. Across these patients, EBUS‐TBMC was attempted on 14 lymph node targets. EBUS‐TBMC was completed on 13 of 14 (92.9%) lymph node stations with a single ‘Failed cryobiopsy’ attempt. Of these 13 lymph node targets, there was a single ‘Incomplete pair’ biopsy with unsuccessful TWC biopsy as the needle tract was lost in a large target following initial *en‐bloc* biopsy (Case 5). There were 12 completed pair biopsies (92.3%) (Tables 1 and 2).

**FIGURE 1:**
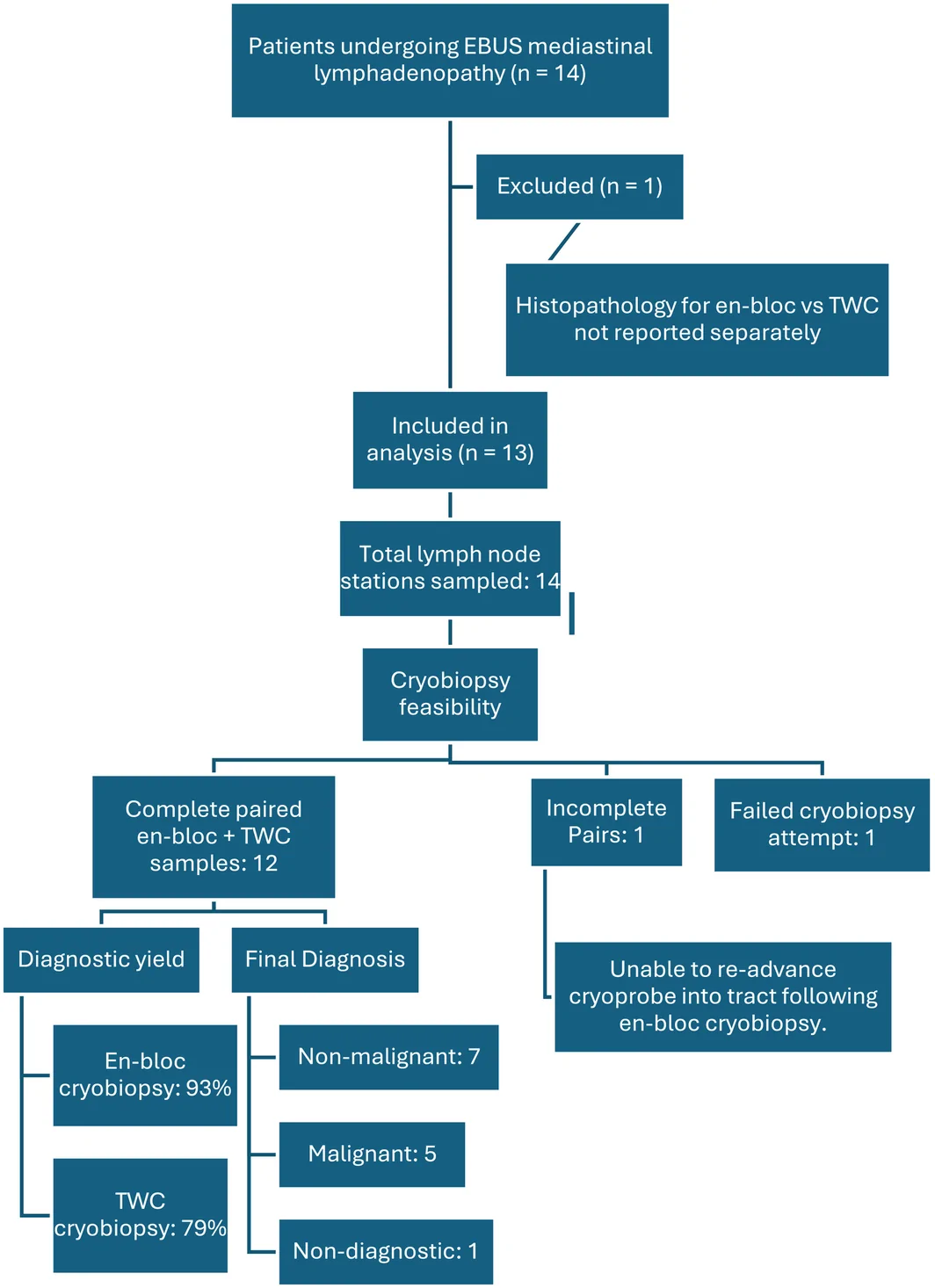
Flow diagram of study and results of primary outcomes.

**TABLE 1:** Sizes and outcomes of specimen samples in en‐bloc cryobiopsies (A).

Cases	A size (mm) first pass	A size (mm) second pass	A outcome
1	3	5	Representative
2	3	3	Representative
3	3	4	Representative
4	Unsuccessful	Unsuccessful	Non‐representative
5	4	Unsuccessful	Representative
5	3	6	Representative
6	4	5	Representative
8	4	4	Representative
9	3	3	Representative
10	4	Unsuccessful	Representative
11	3	3	Representative
12	3	3	Representative
13	2	2	Representative
14	3	3	Representative

**TABLE 2:** Sizes and outcomes of specimen samples in TWC cryobiopsies (B).

Cases	B size (mm) first pass	B size (mm) second pass	B outcome
1	< 1	< 1	Non‐representative
2	2	2	Representative
3	2	2	Representative
4	Unsuccessful	Unsuccessful	Non‐representative
5	Unsuccessful	Unsuccessful	Non‐representative
5	3	3	Representative
6	3	3	Representative
8	4	4	Representative
9	4	4	Representative
10	4	4	Representative
11	3	3	Representative
12	3	3	Representative
13	2	2	Representative
14	3	3	Representative

Among targets with completed paired samples, there was no statistical difference in the size of samples. The median size of ‘En‐bloc cryobiopsy’ was 3 mm (range 2–6 mm) and ‘TWC cryobiopsy’ was similar at 3 mm (range < 1–4 mm, *p* = 0.197). The separate diagnostic yield of ‘En‐Bloc cryobiopsy’ and ‘TWC cryobiopsy’ was similar (93% vs. 79% respectively, *p* = 0.48). Two ‘TWC cryobiopsy’ samples were non‐representative despite representative *en‐bloc* samples.

### 2.6 Secondary Outcomes

Diagnostic yield for EBUS‐TBMC and EBUS‐TBNA was 93% and 46%, respectively. Of the 13 patients included in the study, seven had non‐malignant outcomes, five were malignant and one was non‐diagnostic (presumed reactive lymph node) (Table 3). Chest x‐rays were completed in 10 of 13 cases, with no occurrence of pneumothorax or pneumomediastinum. There were no bleeding events observed.

**TABLE 3:** Outcomes of EBUS‐TBNA, EBUS‐TBMC and final diagnoses.

Cases	EBUS‐TBNA outcome	EBUS‐TBMC outcome	Diagnosis
1	Non‐diagnostic	Non‐necrotising granulomatous inflammation	Sarcoidosis
2	Lymph node only	Non‐necrotising granulomatous lymphadenitis	Sarcoidosis
3	Lymph node only	Reactive lymphoid tissue with anthracotic pigment and epitheliod histiocytes	Benign
4	Malignant	Non‐diagnostic	SCLC
5	Malignant	Malignant	NSCLC
6	Malignant	Malignant	Metastatic PTC
7	Lymph node only	Non‐necrotising granulomatous inflammation	Sarcoidosis
8	Lymph node only	Malignant	NSCLC
9	Malignant	Malignant	Carcinoid
10	Non‐malignant	Non‐necrotising granulomatous lymphadenitis	Sarcoidosis
11	Lymph node only	Granulomatous lymphadenitis with focal areas of degenerative changes	Sarcoidosis
12	Malignant	Malignant	NSCLC
13	Lymph node only	Non‐caseating granulomatous inflammation	Sarcoidosis
14	Lymph node only	Lymph node only	Reactive

Abbreviations: NSCLC, non‐small cell lung cancer; PTC, papillary thyroid cancer; SCLC, small cell lung cancer.

## 3 Discussion

This study demonstrates that EBUS‐TBMC samples removed through the 2.2 mm working channel of the Olympus BF‐UC190F bronchoscope are feasible and yield tissue samples comparable in size to conventional *en‐bloc* cryobiopsy retrieval. Advantages of this method include continuous airway access, which may facilitate earlier recognition and management of bleeding, avoidance of repeated vocal cord passage and reduced procedural time.

With identical freezing times, the differences in specimen sizes did not reach statistical significance. Diagnostic yield of TWC cryobiopsy remained high at 79% and closely aligned with *en‐bloc* cryobiopsy (93%). Apart from cases 1 and 5, where only *en‐bloc* cryobiopsy was conclusive, TWC cryobiopsies appear promising in diagnostic adequacy. As the first case performed of the study, Case 1 was the pilot case and the initial test run of the study, and TWC samples were inadequate. We have elected to include the pilot case despite initial inadequacy to demonstrate rigour and methodical self‐correction. As described above, TWC in Case 5 was non‐diagnostic as the needle tract was lost in a large target following initial *en‐bloc* biopsy.

Histopathological diagnosis, including atypical carcinoid and papillary thyroid carcinoma, was achievable suggesting that modestly smaller specimen size does not compromise diagnostic adequacy. However, the relatively numerically larger sizes of *en‐bloc* specimens may suggest that *en‐bloc* cryobiopsies remain favourable and the small sample population potentially accounts for the non‐significance.

Our study supports previous findings that EBUS‐TBMC achieves higher diagnostic yield than EBUS‐TBNA [1, 2]. A lower diagnostic yield of TBNA was expected with the proportion of non‐malignant diagnoses, particularly granulomatous diseases, where the diagnostic yield of TBNA is established to be inferior to cryobiopsies [1, 2]. However, it is lower than values reported in conventional TBNA literature, which can arise from fewer passes as the focus was placed on cryobiopsies. Furthermore, the absence of major bleeding, pneumothoraces and pneumomediastinum further support the safety profile. Inspection of the bronchoscope following the procedure by Central Sterile Services Department demonstrates no concerns of leak and damage of the bronchoscope, suggesting that the bronchoscope remains safe following TWC cryobiopsies.

Given both approaches are not mutually exclusive, we propose a pragmatic *Hybrid Procedural Strategy* whereby operators perform initial retrievals through the working channel, conduct macroscopic assessment of sample adequacy and, if samples are small or fragmented, perform an *en‐bloc* cryobiopsy if airway conditions remain stable.

Potential disadvantages of TWC cryobiopsy include smaller sample sizes, which may limit ancillary testing. Repeated retrieval of frozen tissue through the working channel may theoretically increase the risk of channel abrasion and bronchoscope damage, although no damage was observed in our study. Additionally, our results may not be generalisable to other Linear EBUS bronchoscopes that have different working channel sizes and/or puncturing angles.

This study has limitations. Sample measurement method was not pre‐defined and one‐dimensional measurements may not fully encompass differences in three‐dimensional volume. A single‐operator series limits generalisability and the small specimen size increases the risk of overestimating effect sizes.

This is the first series to examine the efficacy of TWC as compared to *en‐*bloc cryobiopsies within the same lesion. To date, there is a single non‐comparative study, analysing the feasibility, safety and reproducibility of in‐channel mediastinal cryobiopsy (similar to TWC cryobiopsy of this study) [7]. Larger multicentre studies are required to assess efficacy, non‐inferiority and safety.

In conclusion, this study demonstrates initial feasibility, potential non‐inferiority and safety with no observed major complications of EBUS‐TBMC samples obtained using a 1.1 mm cryoprobe that can be retrieved through the echobronchoscope with a 2.2 mm working channel (*BF‐UC190F*, Olympus Medical, Japan). This approach maintains continuous airway access and may streamline bleeding management while providing tissue samples comparable to conventional *en‐bloc* cryobiopsy.

## Author Contributions

**Jolyn Ng:** writing – original draft, investigation, writing – review and editing, validation, software, formal analysis, data curation, resources. **David Manners:** conceptualization, investigation, methodology, visualization, writing – review and editing, formal analysis, project administration, supervision.

## Ethics Statement

The authors declare that they have obtained appropriate authorisation to access and publish the patients' data from St John of God Midland Public and Private Hospital, authorisation number: St John of God Healthcare Human Research Ethics Committee #1941 and approval date 21 April 2022.

## Conflicts of Interest

The authors declare no conflicts of interest.

## Data Availability

The data that support the findings of this study are available from the corresponding author upon reasonable request.
